# Lipofibromatous Hamartoma of the Median Nerve in a Patient With Recurrent Disease and Preserved Neurological Function: A Case Report

**DOI:** 10.7759/cureus.111740

**Published:** 2026-06-29

**Authors:** Armand Ceniza, Wesley Burrow, Hannah Phan, Kareena Chawla, Gary Schwartz

**Affiliations:** 1 College of Allopathic Medicine, Nova Southeastern University, Fort Lauderdale, USA; 2 Orthopedic Surgery, Dr. Kiran C. Patel College of Allopathic Medicine, Nova Southeastern University, Fort Lauderdale, USA

**Keywords:** carpal tunnel syndrome, fibrolipomatous hamartoma, intraneural lipoma, lipofibromatous, lipofibromatous hamartoma, median nerve tumour, mri- magnetic resonance imaging

## Abstract

Lipofibromatous hamartoma (LFH) is a rare benign tumor typically characterized by fibrofatty infiltration and proliferation within nerve fascicles, often having a predilection for the median and ulnar nerves.

In this case, a 53-year-old woman presented with a painful mass of the left distal forearm that had been present for several years. She had intermittent tingling of the thumb, index, and middle fingers, and a limited range of motion (ROM) in wrist flexion and extension. The percussion test was positive, and magnetic resonance imaging (MRI) revealed a 3 cm lobulated lesion along the ulnar aspect of the flexor carpi radialis tendon. The patient underwent carpal tunnel release and incisional biopsy. Histopathologic examination demonstrated fibroadipose tissue with scattered nerve fibers consistent with LFH.

This patient’s presentation reflects the considerable clinical heterogeneity reported in LFH. A lack of macrodactyly alongside preserved sensory function and reduced ROM exemplifies the unique characteristics of this specific case. The patient had undergone two previous surgical excisions at 18 and 25 years of age, illustrating the challenges associated with the management of this lesion. Operative records from the prior procedure were unavailable. The patient was followed for 11 weeks postoperatively, leading to difficulty in tracking the progression of the lesion.

This case underscores the importance of individualized management strategies that balance symptom relief with preservation of nerve function.

## Introduction

Lipofibromatous hamartoma (LFH) is a rare, benign, slow-growing tumor of peripheral nerves characterized by the infiltration of mature fibroadipose tissue within the epineurium and perineurium, resulting in progressive enlargement of the affected nerve [1,2]. Although the exact etiology remains unknown, LFH is widely regarded as congenital in origin, with the majority of cases manifesting within the first three decades of life [1,3]. The condition was first described by Mason in 1953 and was subsequently identified as a hamartoma by Emmett in 1965 [3,4]. In 1969, Johnson and Bonfiglio introduced the term “lipofibromatous hamartoma,” which remains the most widely accepted nomenclature today [1,5]. Over the ensuing decades, the condition has appeared in the literature under numerous synonyms, including fibrolipoma, neural fibrolipoma, intraneural lipoma, fibrofatty proliferation, and lipomatosis of the nerve, reflecting the historical inconsistency in its classification [1,5,6]. In 2002, the World Health Organization consolidated several of these terms under “lipomatosis of the nerve,” though the term LFH continues to be widely used in clinical practice [6].

LFH most commonly affects the median nerve [2]. The lesion typically presents as a slowly growing, soft, nontender mass on the volar aspect of the wrist, forearm, or hand, with approximately one-fourth to one-third of cases associated with macrodactyly [2,7]. Less frequently, the condition has been reported in the ulnar, radial, sciatic, plantar, and peroneal nerves [2,5]. As of the most comprehensive systematic review to date, 180 cases of median nerve LFH had been documented in the English and French literature as of 2013 [1]. Given its rarity, the true worldwide incidence has not been formally established [8].

We present a case of median nerve LFH in a 53-year-old woman with a longstanding recurrent forearm mass, intermittent neuropathic symptoms, preserved motor and sensory function, and no associated macrodactyly. This case highlights the heterogeneous clinical presentation of LFH, reinforces the diagnostic value of imaging and histopathology in select cases, and contributes to the limited literature guiding management of this uncommon entity.

Emerging molecular evidence has further suggested that LFH, particularly in cases associated with macrodactyly or localized fibrofatty overgrowth, may represent part of a broader spectrum of mosaic overgrowth disorders involving the phosphatidylinositol 3-kinase (PI3K)/AKT signaling pathway rather than an entirely isolated peripheral nerve lesion [9-11]. In a landmark molecular study, Rios et al. identified somatic activating mutations in the PIK3CA gene within affected tissues from patients with macrodactyly and lipofibromatous overgrowth, supporting a pathogenic relationship between LFH-associated macrodactyly and PIK3CA-related overgrowth disorders [9]. Subsequent work by Tian et al. expanded this concept by demonstrating somatic mosaic mutations involving both PIK3CA and AKT1 in patients with isolated macrodactyly, including phenotypes overlapping with Proteus syndrome, thereby suggesting that these lesions exist along a continuum of mosaic segmental overgrowth syndromes [10]. Consistent with these findings, Keppler-Noreuil et al. formally characterized the clinical spectrum of PIK3CA-related overgrowth spectrum (PROS), incorporating fibroadipose overgrowth and congenital segmental enlargement phenotypes that share clinical and molecular similarities with LFH-associated macrodactyly [11]. Although most cases of LFH remain sporadic and localized, these studies support the hypothesis that at least a subset of lesions may reflect localized manifestations of underlying mosaic genetic alterations.

Clinically, LFH most often presents as a slowly enlarging, soft tissue mass in the volar aspect of the forearm or palm, frequently accompanied by symptoms of compressive neuropathy, including pain, paresthesia, and numbness in the distribution of the affected nerve [1,2,7]. The condition tends to follow one of two clinical trajectories: an earlier presentation dominated by macrodactyly (enlargement of one or more digits due to associated fibroadipose overgrowth, reported in approximately 32% of cases) or a later presentation characterized by a forearm or wrist mass with progressive compressive neuropathy symptoms [1,2,12]. Gender distribution is approximately equal, though macrodactyly is more prevalent among females [1,3]. Most patients present before the age of 30, with a reported age range at diagnosis spanning from birth to the seventh decade of life [1,3]. LFH should be strongly considered in patients presenting with median nerve masses accompanied by compressive neuropathy, regardless of whether classic features are present.

Diagnosis of LFH is challenging given its heterogeneous clinical presentation and rarity. Magnetic resonance imaging (MRI) plays a central role in evaluation, demonstrating findings widely considered highly suggestive of LFH, including fusiform nerve enlargement with the characteristic “coaxial cable” appearance on axial sequences and a “spaghetti-like” pattern on coronal images, both reflecting low-signal nerve fascicles interspersed within high-signal fatty tissue [2,12]. While these imaging findings may support a confident diagnosis in classic presentations, histopathologic confirmation remains valuable in atypical, recurrent, or diagnostically uncertain cases, including those involving lesion recurrence, unavailable contrast-enhanced imaging, or atypical patient demographics, particularly when malignant peripheral nerve lesions must be excluded [1,2]. Histologically, LFH is characterized by mature fibroadipose infiltration separating nerve fascicles, with S100 immunostaining highlighting neural elements [1,8].

No universally accepted treatment algorithm currently exists for LFH. Because the fibroadipose infiltration is intimately intertwined with nerve fascicles, complete surgical excision carries a high risk of permanent motor and sensory deficits and is generally not recommended [1,12]. In symptomatic patients, particularly those with compressive neuropathy, nerve decompression (such as carpal tunnel release) is considered the most appropriate intervention to relieve symptoms while minimizing the risk of iatrogenic nerve injury [1,2]. Management should, therefore, be individualized according to symptom severity, degree of functional impairment, extent of nerve involvement, and overall clinical context.

## Case presentation

A 53-year-old, right-hand-dominant woman presented with a painful mass involving the left distal forearm that has been present for two years. The pain was rated 3/10 on an ordinal scale. She previously had a mass removed from the left forearm twice, when she was 18 and 25 years old. She was told that this was a *fatty tumor*. This information was patient-reported, and documentation of this was not available. She had intermittent tingling in the thumb, index, and long fingers. The symptoms were progressive and worsened at night. The past medical history, medications, occupational factors, and comorbidities were noncontributory. On physical examination of the left upper extremity, there was a full active range of motion (ROM) of the shoulder and elbow. There was a very tender 20 x 20 mm soft tissue mass located just radial to the palmaris longus tendon. There was a transverse scar on the volar aspect of the distal forearm from previous surgery (Figure 1).

**Figure 1:**
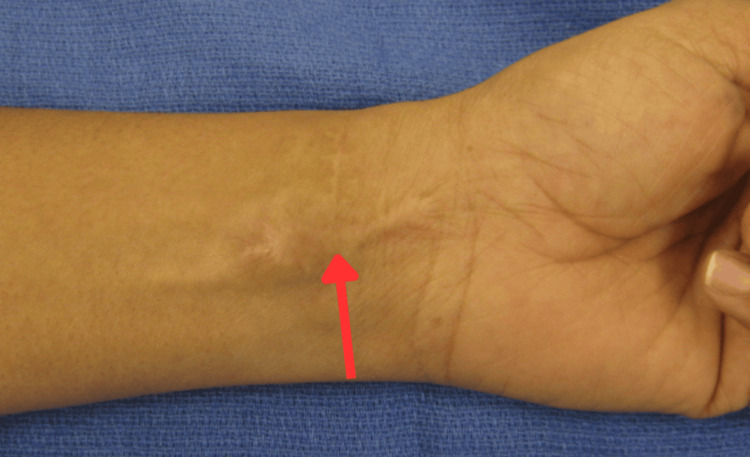
Preoperative appearance of the mass. The soft-tissue mass and previous transverse scar are seen on the volar aspect of the left distal forearm (red arrow).

Tinel’s sign was positive over the mass in the distal forearm. Two-point discrimination was 7 mm on all the digits of the left hand. This was considered to be mildly abnormal. There was no thenar atrophy. Opposition strength was 5/5. Thenar sensation was normal. Active ROM of the left wrist was dorsiflexion 50°, volar flexion 40°, radial deviation 20°, and ulnar deviation 20°. Our patient did not appear to have any clinical features suggesting a mosaic overgrowth pattern. There was no evidence of macrodactyly.

Radiographs of the left distal forearm and wrist did not demonstrate any abnormalities. Radiographs of the hand were not obtained. An MRI scan of the left distal forearm demonstrated a 3 cm, lobular, T1 intermediate-enhancing lesion along the ulnar aspect of the flexor carpi radialis tendon at the level of the distal forearm. The craniocaudal (CC) dimension of the mass was 29 mm. The transverse dimension (TVR) was 14 mm. The anteroposterior dimension was 9 mm. The characteristic coaxial cable appearance of the median nerve was best appreciated on the axial MRI sequence, and the “spaghetti-like” appearance is best seen on the sagittal MRI sequence. The mass was intimately associated with the median nerve before the nerve entered the flexor retinaculum. The median nerve appeared expanded by the mass with thickened nerve fascicles (Figure 2). Electrodiagnostic studies were not performed.

**Figure 2:**
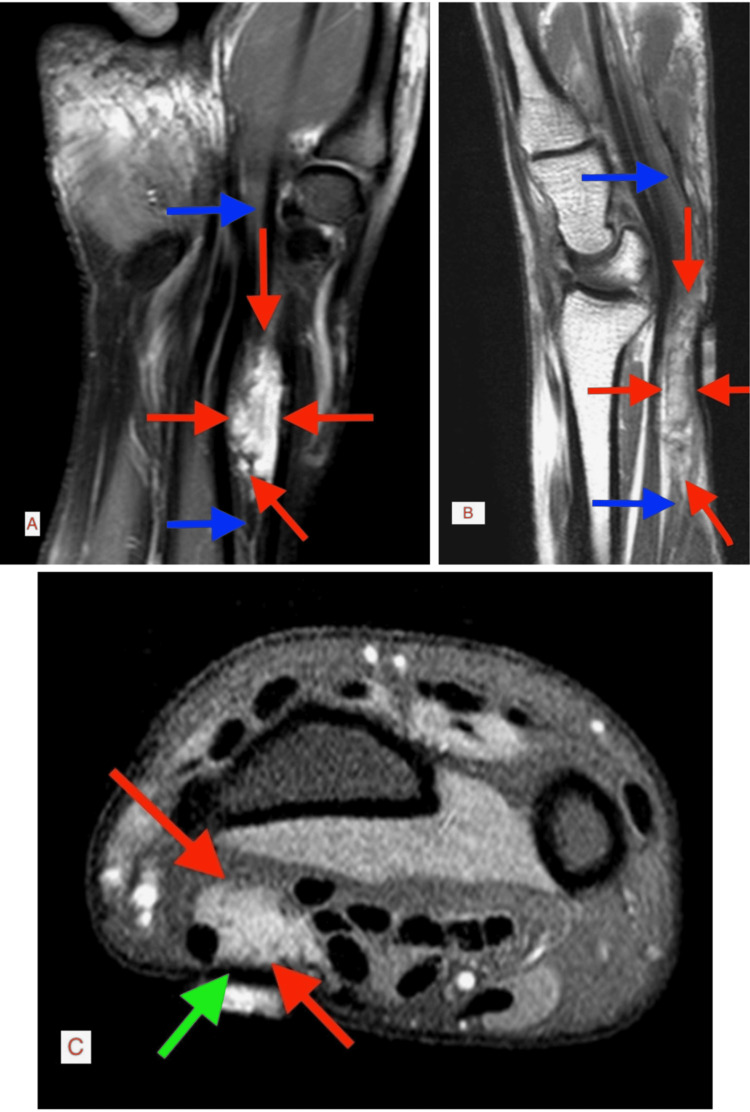
Pre-operative MRI of the left distal forearm and wrist. T1-weighted fat-suppressed and post-contrast coronal (A), sagittal (B), and axial (C) images of the wrist showing an elongated mass (red arrows) inseparable from the median nerve in the distal forearm, just proximal to the flexor retinaculum. The normal median nerve (blue arrows) and the flexor retinaculum (green arrow) are identified. There is associated thickening of the nerve fascicles and mild enhancement on post-contrast images. The dimension of the abnormality in the median nerve is 29 x 14 x 9 mm (CC x TVR x AP). CC, craniocaudal; TVR, transverse; AP, anteroposterior

The patient subsequently underwent surgical exploration, carpal tunnel release, and incisional biopsy. In light of the characteristic MRI findings, a biopsy of the abnormality was indicated due to the patient’s neurologic symptoms and the fact that two previous surgeries were performed. Conservative management was not indicated because of the presence of a mass. The goal of the surgery was nerve decompression, diagnosis, and symptom relief. A tourniquet was utilized during the dissection portion of the procedure, which was 30 minutes. A longitudinal incision was made on the volar aspect of the left distal forearm and wrist. The flexor carpi radialis tendon was identified. The median nerve was identified both proximally and distally. A soft-tissue mass was identified within the substance of the median nerve. The lesion diffusely infiltrated the median nerve, resulting in splaying of nerve fascicles. The carpal tunnel was released (Figure 3).

**Figure 3:**
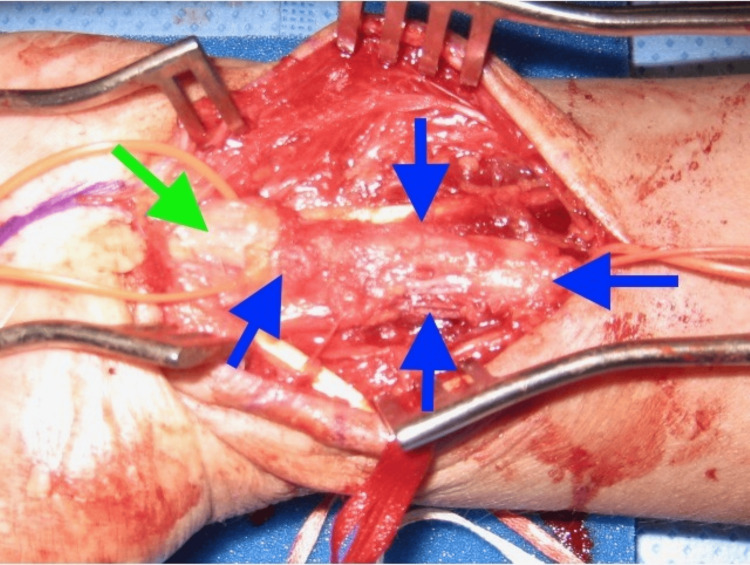
Intraoperative photograph of the median nerve. On the volar aspect of the left distal forearm, the enlarged median nerve (blue arrows) approaching the carpal tunnel, as well as the normal median nerve just proximal to the carpal tunnel (green arrow), are identified.

Since the mass was infiltrating the median nerve, an incisional biopsy of the mass was performed. The pathology report demonstrated fibroadipose tissue with scattered intermingled nerves (S100 stain), consistent with an LFH. The operative reports and imaging from the previous surgeries were unavailable. The actual histologic slides could not be obtained since the pathology facility discarded the slides after 10 years. The patient was subsequently followed up in the office at 1 week, 2 weeks, 5 weeks, and 11 weeks. Her active ROM was dorsiflexion 50°, volar flexion 40°, radial deviation 20°, and ulnar deviation 20°. The surgery improved her pain and paresthesias despite unchanged ROM. Her 2-point discrimination remained at 7 mm on all the digits. This represented preserved function as well as a lack of measurable postoperative improvement. Her pain resolved completely, and the surgical incision was completely healed. The Disabilities Arm, Shoulder, and Hand (DASH) score was not available at that time [13]; however, she was able to partake in all daily activities without any limitation. At that point, the patient was discharged from active care. She was asked to notice any recurrent symptoms, and if they occurred, she was to contact her physician’s office. Table 1 presents the timeline of the patient’s history, surgery, and recovery.

**Table 1:** Timeline of the patient's clinical course. This timeline summarizes the sequence of events starting from the patient’s first surgery until the time she was discharged from active care.

Date	Event
1973	Mass excised for the first time
1980	Mass excised for the second time
November 2008	Initially evaluated by a hand surgeon
December 2008	Magnetic resonance imaging (MRI) of the left distal forearm/wrist obtained
January 2009	Surgery for carpal tunnel release, incisional biopsy of the median nerve lesion
March 2009	Patient discharged from active follow-up

## Discussion

This case highlights the presentation of an LFH involving the median nerve, emphasizing diagnostic challenges and variability in management. Our patient presented with a unilateral mass with median nerve involvement, which is the most commonly affected nerve in LFH. The patient's history suggests that the lesion has been present since at least 18 years old, when she first underwent a surgical procedure for its removal, aligning with the early onset described in literature within the first three to four decades of life [14]. Her initial presentations at that time were not made known, but her current symptoms of pain and intermittent paresthesia likely reflect a common progression of LFH [1,15]. LFH is difficult to diagnose clinically [12] due to its heterogeneous presentation with respect to symptom severity, associated findings, and disease course. The prolonged duration of the lesion preservation of neurological function adds to the heterogeneity of LFH presentation.

In our patient, the follow-up was only 11 weeks. Long-term follow-up is required to determine the risk of recurrence and progression. While our patient demonstrated common clinical presentations of LFH, she also presented with atypical features. LFH has been associated with macrodactyly in up to 32% of cases [1]. Our patient did not exhibit this finding. On clinical examination, our patient exhibited a positive percussion sign, indicating nerve irritation. However, she showed no significant motor or sensory loss. Her preserved motor and sensory functions are important to consider, given that hypesthesia and motor deficits were reported in 28% and 27% of patients, respectively [1]. ROM is typically preserved in LFH [8,16]; however, mild or functional limitations may occur in select cases due to pain, mass effect, or compressive neuropathy [17,18]. Our patient fits into the latter group, as active ROM in her wrist was decreased from the normal 80° of flexion and 75° of extension [19].

Radiographic findings in LFH are often nonspecific but may demonstrate soft tissue enlargement and, in cases associated with macrodactyly, osseous hypertrophy and secondary degenerative changes [1]. These skeletal findings can include cortical thickening and features consistent with early osteoarthritis. In the absence of macrodactyly, however, radiographs are frequently unremarkable. Consistent with this variability, our patient demonstrated no radiographic abnormalities, underscoring the heterogeneity of LFH presentation.

Additional imaging modalities, including ultrasonography and computed tomography (CT), may supplement diagnostic evaluation. Ultrasound-guided steroidal injection may help alleviate the pressure on the median nerve within the carpal tunnel in addition to decreasing the cross-sectional area of the median nerve [20]. MRI has taken a central role in imaging an LFH [1,12], offering both characteristic imaging features and detailed evaluation of nerve involvement. The affected nerve typically demonstrates fusiform or hourglass-shaped enlargement, often with associated displacement of adjacent structures such as flexor tendons and the flexor retinaculum. On axial imaging, the lesion may appear round, oval, or multilobulated, while T1- and T2-weighted sequences reveal low-signal nerve fascicles interspersed within abundant high-signal fatty tissue and fibrous septations. These findings produce distinctive patterns commonly described as *coaxial cable-like* on transverse images and *spaghetti-like* on longitudinal views [2,12], which are considered highly suggestive, almost pathognomonic, for LFH. In addition to facilitating diagnosis, MRI provides valuable preoperative information regarding the extent of nerve involvement.

As helpful as MRI can be, histologic confirmation is the gold standard for a definitive LFH diagnosis. That being said, MRI findings may be highly characteristic, and biopsy may risk nerve injury. Histopathologic evaluation assists in excluding malignant peripheral nerve sheath tumors and other malignant lesions [12]. Histologically, LFH is characterized by the proliferation of mature fibroadipose tissue within the epineurium and perineurium, with nerve fascicles splayed apart and interspersed among the fatty infiltrates. Immunohistochemical staining with S100 protein is used to highlight the scattered nerve fascicles within the lesion, confirming neural origin and supporting the diagnosis. In our patient, the pathology report demonstrated fibroadipose tissue with scattered intermingled nerves on S100 stain, consistent with LFH. The histologic findings were particularly important in this case, given the patient’s recurrent mass and prior surgical history, as the pattern of fibroadipose infiltration within neural tissue distinguished LFH from other soft tissue tumors. Although the original histologic slides from prior surgeries were unavailable, having been discarded after 10 years per institutional policy, the current biopsy provided definitive pathologic confirmation.

The differential diagnosis for LFH includes intraneural lipoma, schwannoma, neurofibroma, ganglion cyst, and malignant peripheral nerve sheath tumors [7,12,17]. Unlike LFH, intraneural lipomas are typically well-encapsulated lesions that displace rather than infiltrate nerve fascicles, whereas LFH demonstrates diffuse fibrofatty infiltration within the epineurium and perineurium [6,7]. Schwannomas and neurofibromas may also present as peripheral nerve masses but generally lack the characteristic interfascicular fatty proliferation and classic MRI findings associated with LFH [7,12]. In our patient, the imaging findings strongly supported the diagnosis preoperatively, while histopathologic confirmation was particularly valuable given the recurrent nature of the lesion and prior surgical history.

Given the infiltrative nature of LFH, management remains controversial with no standardized treatment approach. Management options range from observation in asymptomatic patients to decompression procedures such as carpal tunnel release in those with compressive neuropathy. More aggressive resection or grafting may be considered if a large neurofibroma is diagnosed. Complete resection of the mass has been recommended by some, but this varies by case and is often avoided to prevent severe sensory and functional impairments [1,15]. In patients with compression neuropathy, decompression may be the most appropriate option to relieve symptoms while minimizing the risk of nerve injury [15]. Our case illustrates this clinical dilemma and reinforces the growing consensus that, in the absence of a significant neurological deficit, decompression with biopsy represents a more prudent intervention than aggressive resection.

In our patient, the lesion was present for several decades with prior surgical excisions at ages 18 and 25 in attempts to remove the mass. Given that the patient maintained motor and sensory function despite the recurrence of the mass and its chronicity, a more conservative surgical intervention was indicated. Carpal tunnel release with incisional biopsy was therefore performed to address symptomatic nerve compression and confirm diagnosis. During her post-operative follow-up, her two-point discrimination test remained stable, signifying preserved sensory function. The patient’s history of recurrent disease despite prior excisions and then conservative surgery (carpal tunnel release) emphasizes the infiltrative nature of LFH and its challenges with selecting appropriate treatment options. Although this report is limited by its single-patient design and short follow-up duration, it contributes to the growing body of literature supporting nerve-sparing surgical approaches for patients with preserved neurological functions. Given that LFH is rare and may be misdiagnosed, clinicians must consider LFH in patients with median nerve masses and compressive neuropathy symptoms, even if they lack common presentations such as macrodactyly or significant neurologic deficits. Tumors such as schwannomas, neurofibromas, lipomatous lesions, and tenosynovial lesions may be confused with LFHs. 

Limitations of this case should be considered. The details of her past medical and surgical history were unavailable, which limited the ability to track her initial diagnosis and disease progression over time. Additionally, the patient's family history was unavailable and unknown, preventing assessment of potential genetic relationships to LFH. Genetic testing was not performed in this patient because the results were unlikely to alter clinical management. Emerging evidence linking LFH to mosaic PI3K/AKT pathway alterations may further explain her clinical presentation and disease progression. The patient’s long-term outcomes, recurrence, symptom relief, and progression also remain unknown, as postoperative follow-up was limited to 11 weeks. Further longitudinal studies are needed to better characterize these outcomes. Another potential limitation is that ultrasonography and electrodiagnostic studies were not performed on our patient, limiting our ability to determine the degree of the median nerve entrapment. As a single case report, these findings are not generalizable to the larger population.

A table of the selected articles, listed by author and study title, is presented in Table 2.

**Table 2:** Literature review table. The case studies and series included in our literature review are listed by authors, the title of their study, and a summary of findings. LFH, lipofibromatous hamartoma; MRI, magnetic resonance imaging; CTS, carpal tunnel syndrome

Ref #	Author(s)	Article	Summary/Objectives	Findings
[2]	Al-Jabri et al. (2010)	Lipofibromatous Hamartoma of the Median Nerve	-Case report of a 24-year-old patient with LFH of the median nerve causing secondary carpal tunnel syndrome -Clinical presentation included worsening dysesthesia and paresthesia affecting his right thumb and index finger. -Tinel’s sign and Phalen’s test were positive.	-Median nerve LFH presented with paresthesia and positive provocative testing; MRI showed characteristic cable-like enlargement, and decompression was favored over excision to reduce nerve injury risk.
[3]	Silverman and Enzinger (1985)	Fibrolipomatous Hamartoma of Nerve: A Clinicopathologic Analysis of 26 Cases	-Case series discussing 19 cases of LFH of peripheral nerves without macrodactyly and 7 cases with macrodactyly -Aims to highlight the presentation, histology, and association with macrodactyly to characterize diagnosis and management of LFH	-A strong but not universal association between LFH and digit enlargement may suggest congenital or developmental abnormality relationships. -LFH commonly involved the hand and showed fibrofatty infiltration of the epineurium/perineurium; neurologic symptoms were related to compression, and conservative management often had a benign course.
[4]	Emmett (1965)	Lipomatous Hamartoma of the Median Nerve in the Palm	-One of the earliest reported cases of LFH involved the median nerve. -A 6-year-old girl with progressively worsening soft swelling of the left hand for three years	-Early median nerve case describing a slowly enlarging fibrofatty nerve lesion with compressive features, supporting LFH as a benign median nerve process. -Termed the lesion "hamartoma" instead of congenital lipoma.
[5]	Johnson and Bonfiglio (1969)	Lipofibromatous hamartoma of the median nerve.	-Case report describing a rare tumor-like lesion involving the median nerve.	-Indicates excision of all or part of the mass certainly produces a significant neurologic deficit in the hand and is thus contraindicated.
[7]	Toms et al. (2006)	Lipofibromatous Hamartoma of the Upper Extremity: A Review of the Radiologic Findings for 15 Patients	-Analysis of radiographic characteristics of LFH affecting upper limb peripheral nerves in 15 patients.	-In 15 upper-extremity LFH cases, MRI demonstrated a characteristic coaxial cable/spaghetti-like appearance. - Imaging findings were often sufficient to establish the diagnosis without the need for biopsy.
[8]	Shekhani HN, Hanna T, Johnson JO (2016)	Lipofibromatous Hamartoma of the Median Nerve: A Case Report	-Case report of LFH in an 8-year-old child. Discusses epidemiology, diagnosis, imaging details, and treatment options for this condition.	-Pediatric median nerve LFH showed classic MRI findings and histology with mature adipose/fibrous tissue separating nerve fascicles.
[15]	Louis et al. (1985)	Lipofibromas of the Median Nerve: Long-Term Follow-Up of Four Cases	-Four cases of LFH on the median nerve with long-term follow-up (average 11 years). -Progressive compromise of median nerve function.	-Long-term median nerve LFH cases showed variable progression and neurologic compromise -Initial treatment considerations should be directed toward biopsy and alleviation of the compression neuropathy. Uncertain of whether microsurgical debulking procedures are worth it.
[16]	Yoo et al. (2023)	10-Year Clinical Follow-Up after Decompression of Lipofibromatous Hamartoma of the Median Nerve in a 3-Year-Old Patient: Case Report and Review of the Literature	-Reports a case of median nerve LFH in a 3-year-old female patient	-Median nerve LFH treated with carpal tunnel release and epineurolysis had no recurrence or motor/sensory deficit at 10-year follow-up.
[17]	Michailidis et al. (2024)	Two Cases of Fibrolipomatous Hamartomas of the Median Nerve: A Rare Entity With Pathognomonic MRI Features	-Presents two cases of median nerve LFH. -Highlights the pathognomonic MRI features of LFH. -Emphasizes the role of MRI in noninvasive diagnosis and management.	-MRI demonstrated characteristic “coaxial cable” (axial) and “spaghetti string” (coronal) appearances in both cases. -Conservative management was successful in both cases due to the absence of significant neurologic deficits.
[18]	Senger et al. (2014)	Fibrolipomatous Hamartoma of the Median Nerve: A Cause of Acute Bilateral Carpal Tunnel Syndrome in a Three-Year-Old Child: A Case Report and Comprehensive Literature Review	-Case report of the first published instance of acute bilateral CTS secondary to bilateral FLH of the median nerves in a pediatric patient (3-year-old boy), presenting as incessant crying	-Bilateral carpal tunnel release with biopsy confirmed LFH and resulted in full recovery, supporting decompression over radical interfascicular dissection.

## Conclusions

This case illustrates the diagnostic and therapeutic complexity inherent to LFH, a rare peripheral nerve lesion whose heterogeneous presentation, including atypical features such as advanced patient age, absence of macrodactyly, and decades of recurrent disease, can challenge even experienced clinicians. MRI remains the cornerstone of initial evaluation, with its characteristic imaging features capable of supporting the diagnosis, while histopathologic confirmation can provide diagnostic confirmation and assist in excluding alternative or malignant pathology when clinically indicated.

This case, with prior reports, underscores the need for individualized surgical decision-making in patients with LFH. The infiltrative nature of LFH may make complete excision challenging and may increase the risk of neurologic morbidity. This case supports consideration of nerve-sparing decompression as a potential management strategy in selected patients with preserved motor and sensory function. Interpretation of the findings of this case is limited by the single case report and the relatively short duration of follow-up. Clinicians could therefore maintain a broad index of suspicion for LFH in patients presenting with median nerve masses and compressive neuropathy, including cases lacking classic features such as macrodactyly. Treatment planning may be based on the patient’s functional condition, disease course, and desired outcome.
